# Laparoscopic cholecystectomy with common bile duct exploration for choledocholithiasis in a patient with situs inversus totalis - Case report and review of literature

**DOI:** 10.1016/j.ijscr.2025.111238

**Published:** 2025-03-29

**Authors:** Sai Krishna, Kalayarasan Raja, Biju Pottakkat

**Affiliations:** Department of Surgical Gastroenterology, Jawaharlal Institute of Postgraduate Medical Education and Research (JIPMER), Puducherry, India

**Keywords:** Laparoscopic common bile duct exploration, Situs inversus totalis, Choledocholithiasis, Mirror image transposition, Case report

## Abstract

**Introduction and importance:**

Laparoscopic common bile duct (CBD) exploration with cholecystectomy in patients with situs inversus totalis (SIT) is technically demanding and warrants comprehensive knowledge of the mirror image anatomy along with laparoscopic orientation of the left upper quadrant.

**Case presentation:**

In this case report, we present a female patient who presented with pain in the left hypochondrium, yellowish discoloration of the eyes since 2 months. She was evaluated, diagnosed to be a case of SIT with cholelithiasis and choledocholithiasis in cholangitis and endoscopic attempts for stone clearance failed. She subsequently underwent laparoscopic common bile duct exploration (LCBDE) with cholecystectomy. Per-operative, cystic duct was short and a choledochoscope was utilized for bile duct clearance.

**Clinical discussion:**

Laparoscopic surgery in patients with SIT is complex, technically challenging and requires a in depth knowledge of remodeled anatomy. We noted a change in the relation of portal vein with CBD as in the portal vein was postero-lateral to bile duct rather than being completely posterior. The key here is to stay close to the common bile duct which can avoid confusion and inadvertent injury to vital structures in the hepato-duodenal ligament.

**Conclusion:**

Laparoscopic cholecystectomy and CBD exploration in situs inversus is extremely challenging and the surgeon should be aware of the ergonomics and practice to operate laparoscopically with the opposite hand I.e the non-dominant hand in right-handed surgeon. Thus, laparoscopic cholecystectomy with LCBDE in situs inversus totalis is safe and feasible but demands technical difficulties.

## Highlights

• Surgical management of biliary tract stones in patients with Situs inversus totalis (SIT) is technically complex.
• A hepato-biliary surgeon should be aware of the altered anatomy since endoscopic management in these patient of SIT with choledocholithiasis often fails due to altered anatomy of duodenum.
• This report highlights the challenges faced during laparoscopy in these patients and how to overcome them.

## 1 Introduction

Laparoscopic common bile duct exploration (LCBDE) with cholecystectomy is a widely accepted technique for managing choledocholithiasis, especially in patients with failed Endoscopic Retrograde cholangiography (ERC) and stone extraction [1]. The difficulty in ERC and stone retrieval may result from large, multiple or impacted stones or altered anatomy of the duodenum and the ampulla [2]. One such rare condition of altered anatomy is situs inversus totalis (SIT), where there is mirror image transposition of abdominal and thoracic organs. SIT, an autosomal recessive congenital disorder, was first reported by Kruchenmeister in 1824 [3]. While the occurrence of biliary tract disorders in SIT is comparable to that of the general population, it poses a significant diagnostic and management challenge due to altered anatomy [4]. In this report, we describe a SIT patient with cholelithiasis and choledocholithiasis with a history of failed ERC managed with laparoscopic cholecystectomy and CBD exploration and a brief review of the literature. This work has been reported in line with the SCARE criteria [5].

## 2 Case report

A 56 year old female who was a known diabetic and hypertensive on regular medication, presented with dull aching type of pain in the left hypochondrium radiating to back and associated with occasional non bilious vomiting for the past 2 months which was managed by over the counter analgesics. She had history of yellowish discoloration of eyes with passage of dark yellow urine and fever of multiple spikes for 5 days. On examination she was icteric, well hydrated and abdominal examination revealed mild tenderness in the left hypochondrium. She was initially admitted and evaluated by the team of Medical Gastroenterology. Her blood investigations are depicted in the Table 1.

**Table 1:** Preoperative & postoperative blood investigations of the study patient.

Haemoglobin - 13.3 g/dl (13.1–17.2 g/dL)	Blood urea – 48 mg/dl (17–43 mg/dl)	Direct bilirubin - 4.59 mg/dl (0.03–0.18 mg/dL)	Gamma glutamyl transferase - 499 U/L (5–40 U/L)	INR - 1.11 (0.8–1.1)
Preoperative blood investigations of the study patient -				
TLC - 17,050 cells/cumm (4000–11,000 cells/cumm)	Serum creatinine - 1.16 mg/dl (0.6–1.2 mg/dl)	AST - 380 (0–35 IU/L) ALT - 819 (0–35 IU/L)	Serum amylase - 19 U/L (22–80 IU/L)	
Platelet - 3,60,000 cells/cumm (150000–400,000 cells/cumm)	Total bilirubin −7.35 mg/dl (0.3–1.2 mg/dl)	Alkaline phosphatase - 436 IU/L (30–120 IU/L)	Prothrombin time - 12.1 s (10–13 s)	

Postoperative blood investigations of the study patient -				
Haemoglobin - 11.1 g/dl (13.1–17.2 g/dL)	Blood urea – 22 mg/dl (17–43 mg/dl)	Direct bilirubin - 0.35 mg/dl (0.03–0.18 mg/dL)	Gamma glutamyl transferase - 49 U/L (5–40 U/L)	INR - 0.93 (0.8–1.1)
TLC - 10,800 cells/cumm (4000–11,000 cells/cumm)	Serum creatinine - 0.61 mg/dl (0.6–1.2 mg/dl)	AST - 18 (0–35 IU/L) ALT - 21 (0–35 IU/L)	Serum amylase - 39 U/L (22–80 IU/L)	
Platelet - 2,23,000 cells/cumm (150000–400,000 cells/cumm)	Total bilirubin −1.09 mg/dl (0.3–1.2 mg/dl)	Alkaline phosphatase - 158 IU/L (30–120 IU/L)	Prothrombin time - 10.1 s (10–13 s)	

**Fig. 1:**
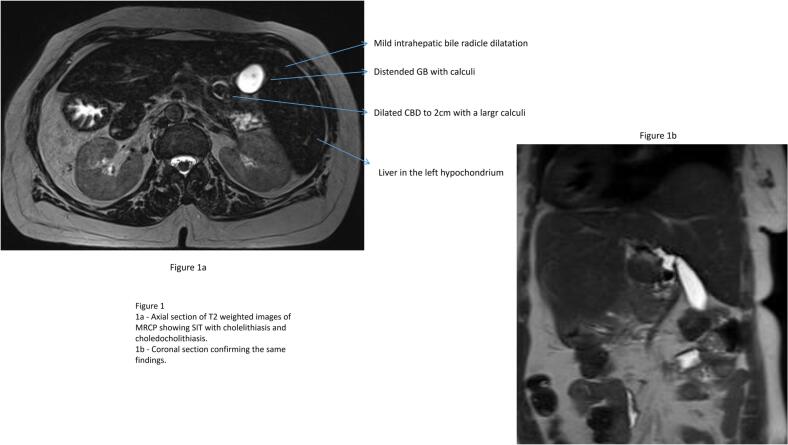
MRCP axial and coronal section showing situs inversus with cholelithiasis and choledocholithiasis.

An ultrasonogram abdomen revealed liver in the left hypochondriac and epigastric region with normal echoes and no focal lesions, Gall bladder calculi of 1 cm with normal wall thickness and sonographic positive Murphy's sign. The common bile duct could not be visualised due to excessive bowel gas. An MRCP revealed features of situs inversus with distended gall bladder with large calculus of 1.5 cm and dilated CBD of 2 cm with mid CBD calculus of 1.8 cm with dilated intra hepatic biliary radicles (Fig. 1). She was eventually diagnosed with situs inversus totalis, cholecystolithiasis, and choledocholithiasis with obstructive jaundice in cholangitis. In view of the cholangitis, she received parenteral antibiotics and was planned for ERC, stone retrieval and biliary stenting. However, ERC was attempted and failed twice due to altered duodenal anatomy and difficult manipulation. Subsequently, she was referred to our team of Surgical Gastroenterology and a decision for surgical intervention was made.

### 2.1 Surgical technique

**Fig. 2:**
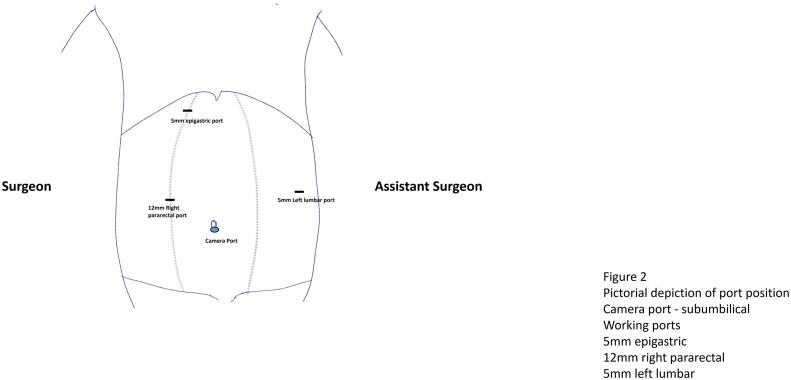
Pictorial depiction of port placement.

**Fig. 3:**
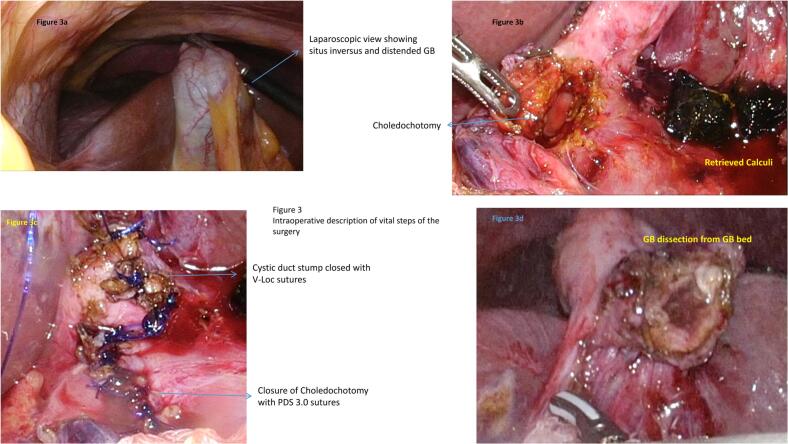
Intraoperative findings of surgical procedure.

Under General anaesthesia, the ports were placed as depicted in the Fig. 2. A sub-umbilical 12 mm port was inserted by open technique and pneumoperitoneum was created with CO2 and 3D laparoscope was introduced. Left lumbar 5 mm port, right para-rectal port 12 mm port and 5 mm epigastric ports were placed under direct vision. The gallbladder was retracted upwards and laterally. Adhesion was released. Posterior and anterior peritoneal layers were dissected out and cystic artery was identified and ligated. CBD was opened 1 cm below the cystic duct insertion. Two large stones of size 1 cm were removed and thorough saline irrigation was given. A choledochoscope was passed in to CBD and stone clearance was confirmed. Cystic duct was short and dilated. Cystic duct was divided at infundibulum and suture closed with barbed absorbable suture material (3–0). CBD was repaired with PDS 3–0 sutures in intermittent fashion. Gallbladder was dissected from the liver bed and delivered out through the umbilical port in retrieval bag with the CBD stone. One 16 French suction drain was placed in the subhepatic space (Fig. 3). She was started on oral sips on Post-operative Day (POD) 1 and further escalated to soft diet on the same day. Her drain output remained around 20-30 ml/ day (serous), hence a decision was done to retain the drain. Her blood investigations at the time of discharge were as depicted in the Table 1. Her port sites remained healthy and she was discharged in a stable condition with drain in situ. On further follow up after 1 week, the drain output was reduced to 10 ml/day and eventually drain was removed. The histopathological examination of the gall bladder revealed features of chronic cholecystitis with cholelithiasis.

## 3 Discussion

Laparoscopic cholecystectomy has increasingly become the standard treatment for both uncomplicated and complicated biliary stone disease, with a growing number of articles discussing its widespread adoption [6]. However, in cases of SIT, where the visceral organs are mirrored from their usual positions, laparoscopic cholecystectomy with or without CBD exploration exploration present unique technical challenges due to the reversed anatomical orientation. Surgeons must exercise particular caution to avoid accidental injury in this unfamiliar surgical landscape [7,8]. Our literature review identified a total of 9 reported cases of laparoscopic CBD exploration in patients with SIT for choledocholithiasis [[9], [10], [11]]. Of these, preoperative ERCP was attempted but failed in five cases, predominantly due to the altered anatomy of the duodenum and ampulla, making cannulation difficult [12,13]. Intraoperative cholangiography (IOC) and the use of a choledochoscope were employed in two and three cases, respectively, leading to successful clearance of CBD stones. Most reports indicated that the surgeon operated from the right side, with one exception where the surgeon positioned themselves between the patient's legs [14]. The standard four-port technique was used in all cases except for one, which utilized a multichannel port to perform a single-incision laparoscopic CBD exploration [15]. Other intraoperative parameters and postoperative outcomes are depicted in Table 2.

**Table 2:** Preoperative and intraoperative parameters of the case reports describing laparoscopic CBD exploration for choledocholithiasis in patients with situs inversus totalis.

Author/year	Preoperative co-morbidities	Preoperative ERCP intervention	Intraoperative application of IOC or Choledochoscope	Number of ports	Technique applied for CBD exploration	Length of hospital stay (days)
Chiu BY, 2022	Hypertension, diabetes and chronic kidney disease	No	IOC	Multichannel port	Transcystic approach with wire basket retrieval	3
Simkhada, 2021	Hypertension and chronic kidney disease	Yes	Choledochoscope	4	Choledochotomy with primar closure	Not specified
Takalkar, 2018	No comorbidities	Yes	None	4	Choledochotomy with choledochoduodenostomy	Not specified
Senthilnathan, 2017	No comorbidities	Yes	IOC	4	Choledochotomy with choledochoduodenostomy	7
Liu, 2017	No comorbidities	No	Not specified	4	Not specified	3
Han, 2012	No comorbidities	Yes	Choledochoscope	4	Choledochotomy with primary closure	Not specified
Tai, 2004	Coronary heart disease	Yes	Choledochoscope	4	Choledochotomy with primary closure	Not specified
Kang, 2004	Cardiac arrhythmias	No	Choledochoscope	4	Choledochotomy with T tube insertion	Not specified
Our case report, 2023	Hypertensive and diabetic	Yes	Choledochoscope	4 (Modified)	Choledochotomy with primary closure	3

Our patient presented with cholangitis and was initially treated with intravenous antibiotics, with a plan for ERC and stone removal. However, due to a failed ERC, we opted for an operative management. During the procedure, we made a slight modification to the standard port placement by introducing a right pararectal port specifically for CBD exploration. Our modified technique involved using both the right pararectal port and a left lumbar port for the exploration of the CBD. A choledochoscope was employed for CBD clearance, followed by primary closure of the choledochotomy. A key principle we adhered to, and which should be considered a critical step in such cases, is to avoid excessive dissection near the CBD, as the altered anatomy in SIT can significantly alter the anatomical relationships of the portal vein, hepatic artery, and CBD within the hepatoduodenal ligament. In our case, for example, the portal vein was observed to be more anteriorly positioned and was crossing the distal portion of the CBD (Fig. 3). By following this principle, the risk of inadvertent injuries can be minimized in patients with altered anatomy.

## 4 Conclusion

Laparoscopic cholecystectomy and CBD exploration in patients with situs inversus poses significant challenges due to the mirrored anatomical orientation. Surgeons must be mindful of the altered ergonomics and practice operating with their non-dominant hand, particularly for right-handed surgeons, since the usual orientation is reversed. Despite these difficulties, the fundamental principles of surgery remain unchanged. Therefore, while laparoscopic cholecystectomy with laparoscopic CBD exploration in situs inversus totalis is safe and feasible, it requires overcoming notable technical challenges.

## Author contribution

Study concept/design: R Kalayarasan/Biju Pottakkat.

Data collection: Sai Krishna.

Data analysis: Sai Krishna.

Writing the paper: R Kalayarasan.

Final check and corrections: R Kalayarasan/Biju Pottakkat.

## Consent

Written informed consent was obtained from the patient for publication and any accompanying images. A copy of the written consent is available for review by the Editor-in-Chief of this journal on request.

## Ethical approval

Ethical approval for this case report was provided by ethical committee of JIPMER, Puducherry on 10 November 19, 2024.

## Guarantor

Sai Krishna.

## Funding

No funding was provided.

## Declaration of competing interest

No conflict of interest.
